# Geographic barriers and Pleistocene climate change shaped patterns of genetic variation in the Eastern Afromontane biodiversity hotspot

**DOI:** 10.1038/srep45749

**Published:** 2017-04-11

**Authors:** Mario Mairal, Isabel Sanmartín, Alberto Herrero, Lisa Pokorny, Pablo Vargas, Juan J. Aldasoro, Marisa Alarcón

**Affiliations:** 1 Real Jardín Botánico (RJB-CSIC), 28014 Madrid, Spain; 2 Royal Botanic Gardens, Kew (RBGK), Richmond, Surrey, TW9 3DS, UK; 3 Instituto Botánico de Barcelona (IBB-CSIC-ICUB), 08038 Barcelona, Spain; 4 Universidad Rey Juan Carlos, Móstoles, Spain

## Abstract

The Eastern African Afromontane forest is getting increased attention in conservation studies because of its high endemicity levels and shrinking geographic distribution. Phylogeographic studies have found evidence of high levels of genetic variation structured across the Great Rift System. Here, we use the epiphytic plant species *Canarina eminii* to explore causal explanations for this pattern. Phylogeographic analyses were undertaken using plastid regions and AFLP fragments. Population genetic analyses, Statistical Parsimony, and Bayesian methods were used to infer genetic diversity, genealogical relationships, structure, gene flow barriers, and the spatiotemporal evolution of populations. A strong phylogeographic structure was found, with two reciprocally monophyletic lineages on each side of the Great Rift System, high genetic exclusivity, and restricted gene flow among mountain ranges. We explain this pattern by topographic and ecological changes driven by geological rifting in Eastern Africa. Subsequent genetic structure is attributed to Pleistocene climatic changes, in which sky-islands acted as long-term refuges and cradles of genetic diversity. Our study highlights the importance of climate change and geographic barriers associated with the African Rift System in shaping population genetic patterns, as well as the need to preserve the high levels of exclusive and critically endangered biodiversity harboured by current patches of the Afromontane forest.

The Afromontane Floristic Region^1^,^2^ refers to a plant assemblage where a series of isolated highland forested areas are separated by lowland vegetation, and form an “archipelago-like” centre of endemism in the mountains of East and West Africa. The eastern part of this floristic region has been singled out as a biodiversity hotspot and is known as the Eastern Afromontane biodiversity hotspot^3^ (EABH) (Fig. 1a). The Eastern African “sky islands” (including both high plateaus and isolated mountain peaks), are sometimes referred to as the “Galapagos of Africa”^4^, and contain such flagship species as the mountain gorilla, the gelada, the Ethiopian wolf, or the coffee tree. These mountain ranges exhibit extraordinary levels of species endemism, for example, out of the > 10.000 plant species found in this region (7600 vascular plants), about one third are endemic^5^. This pattern of endemism has been attributed to long-term climatic stability, favouring lineage diversification and species accumulation over time^6^. The EABH is also one of the most threatened ecosystems in Africa^5^. Ethiopian montane forests, which two hundred years ago^7^,^8^ covered up to 35% of the Afromontane areas, are estimated to have had their range dramatically reduced by human activity in the last century^9^,^10^.

Geographically, the Eastern Afromontane region is divided longitudinally by the Great Rift System, a 4830 km north-south fissure whose terrestrial section extends from Djibouti to Mozambique. This orogenic fault marks the line along which the African Plate is splitting into two tectonic plates, the Somalian Plate and the Nubian Plate (Fig. 1a). The formation of the Rift commenced 31 Ma, as a result of the collision of the African and Arabian Plates with Eurasia; as extension continues, with ongoing volcanic activity^11^, complete breakup of the Somalian Plate is predicted to occur within 10 million years, with a new ocean basin originating where shallow lakes can now be found^12^. The northern part of the Rift runs south from the Afar Triangle and is surrounded by the Ethiopian Highland Plateaus (Fig. 1a): the Abyssinian massif (northwest Plateau) and the Harar massif (southeast Plateau). In the south tropical East Africa, the Rift System is divided into two arcs: the Eastern Gregory Rift and the Western Albertine Rift, which join at the northern end of Lake Malawi, where the Southern Mountain Sky-Islands (SMSI) lie (Fig. 1a). Due to its elevation and extent, this latter region is sometimes known as the “Roof of Africa”. Because of its geographic position, the Rift System is considered one of the most isolated mountain ranges in the world, compared to other tropical floras^13^.

The Afromontane vegetation is divided into three altitudinal belts, each with unique floristic composition^14^,^15^,^16^: the lowermost Afromontane forest (1300–3000 m), the ericaceous belt (3000–4100 m), and the uppermost Afroalpine zone ( > 3550 m). These vegetation belts are not continuous in their distribution but appear divided into forest patches isolated from one another by environmental barriers (Fig. 1b), including extensive savannah areas, semi-deserts, lowland forests (in the past), or (at present) an agricultural matrix of cultivated lands and forestry plantations^1^,^17^,^18^,^19^,^20^. When compared to other mountain systems, the Afromontane vegetation is considered to be rather uniform in composition and is formed by a large number of broad-range species^13^; though given the fragmented nature of the Afromontane region, with numerous forest patches, some of these widespread species are likely to include cryptic diversity^21^.

Interest on geographic patterns of genetic variance in Eastern African species has increased in recent years. Many of these phylogeographic studies (focusing on a diverse array of organisms, Table 1) have reported high levels of genetic variation structured with respect to the Great Rift Valley^8^,^19^,^22^, suggesting it has acted as a dispersal barrier for an extended period of time.

The complex phylogeographic structure found in African highland species has also been explained by two non-mutually-exclusive dispersal-migration scenarios (Fig. 1b.): I) the “mountain-forest bridge” hypothesis postulates that current patterns might be explained by short-range or stepping-stone dispersal (SSD) between adjacent mountain ranges on each side of the Rift Valley, which would have been promoted by a more extensive forest coverage during Plio-Pleistocene warm and humid interglacial periods^8^,^17^,^23^,^24^,^25^; II) the “long-distance dispersal” hypothesis postulates that long-distance dispersal (LDD) events among isolated mountain populations during Pleistocene glacial periods are responsible for the observed phylogeographic patterns^18^,^19^,^26^. These two hypotheses are not mutually exclusive. For example, proponents of the SSD forest bridge hypothesis^17^ suggested LDD migration among some isolated mountain ranges^18^. A requisite to discriminate between these two scenarios is a temporal framework. Most Eastern African phylogeographic studies in plants do not include time estimates for colonization events and, therefore, do not allow discriminating between the mountain-forest bridge and long-distance dispersal hypotheses. This is further aggravated by the difficulties intrinsic to collecting material of widespread Afromontane species, due to the complex geographic landscape and the recent political instability^27^ (see below).

Here, we examine support for these two hypotheses, and the role of the Rift Valley as a long-term dispersal barrier, by reconstructing patterns of genetic variation in *Canarina eminii* Asch. & Schweinf, a strictly Afromontane species with a geographic distribution extending from the Ethiopian massifs to the SMSI in the northern end of Lake Malawi^28^, along the Rift Valley. This species grows mostly as a twig epiphyte on trees, mainly of genera *Hagenia* J.F.Gmel., *Conopharyngia* G. Don, and *Afrocarpus* (J. Buchholz & E.G. Gray) C.N. Page^28^, all characteristic elements of the Afromontane forest belt^28^. Little is known about the reproductive biology of *C. eminii*, except that the species is pollinated by sunbirds (unpublished field observations) and exhibits floral traits that have been associated with this type of ornitophilous pollination^29^ (e.g., large orange-red coloured flowers producing abundant diluted nectar). Fruits are fleshy, with the seeds enclosed in a sticky sweet jelly, suggesting endozoochorous dispersal (e.g., by monkeys^30^). Mairal *et al*.^31^ recently reconstructed the biogeographic history of *Canarina* L., a small genus of three species within tribe Platycodoneae (family Campanulaceae). This genus exhibits a wide disjunct distribution across North Africa and probably originated from a Central Asian ancestor, which arrived to Eastern Africa in the Mid Miocene (13.7 Ma) through the Arabian Plate^31^. The lineage leading to the Eastern African species *C. abyssinica* diverged first, followed by the split between *C. eminii* and the endemic Canarian species *C. canariensis*^31^ in the Late Miocene (c. 7 Ma). Divergence within *C. eminii* was traced back to 1.76 Ma^31^ (southern range of the Great Rift Valley and followed by subsequent colonisation of the northern range). However, the intraspecific sampling in the study by Mairal *et al*.^31^ was limited, which prevented analysis of patterns of genetic variation or examination of the processes population divergence in *C. eminii*.

In this study, we reconstruct the phylogeographic history of *C. eminii* among and within populations. Our aims were to: i) describe the geographic distribution of genetic variation within this species; ii) examine the role of the Rift Valley as a long-term dispersal barrier; iii) understand how Pleistocene climatic fluctuations might have affected population ranges, especially in relation to the “mountain-forest bridge” hypothesis and the role of isolated “sky islands”; and iv) provide insights into the phylogeography of an Afromontane epiphytic species. Since the epiphytic growth of this species is tied to dominant tree species of the Afromontane forests, it makes *C. eminii* a good case study for tracing the history of fragmentation and expansion of forest coverage in this region.

## Results

### Haplotype analyses

In order to examine patterns of genetic variation within *C. eminii*, we sequenced three highly variable plastid (pDNA) regions covering the entire distributional range of this species (see Materials and Methods). We generated 237 new sequences from 79 individuals: *rpl*32-*trn*LUAG (79 sequences), *trn*SGCU–*trn*GUCC (79 sequences), and *pet*B1365–*pet*D738 (79 sequences). The number of nucleotide sites ranged from 648 in *trn*SGCU–*trn*GUCC, 834 in *pet* B_1365_–*pet*D_738_, to 963 in *rpl*32-*trn*L_UAG_. The concatenated matrix consisted of 79 sequences of *C. eminii* with 2445 nucleotide sites. The haplotype network recovered by TCS identified nine haplotypes (H1 to H9; Fig. 2a), divided into two main groups separated by five substitutions (Fig. 2b): one group east of the Rift Valley (Harenna Forest, Agere Maryam, and Aberdare Mts.), and a second group including populations of the Abyssinian massif, west of the Gregory Rifts, the Albertine Rift, and the SMSI. All haplotypes, save H5 and H7, were restricted to one population. Lineage divergence time estimation in the Bayesian software BEAST (Fig. 3, Supplementary Fig. S2.1) supported a similar geographic structure. The first population divergence event (1.92 Ma, 95% high posterior density (HPD) credibility interval (CI): 0.63–3.82 Ma) separated the populations situated at each side of the Rift Valley, with subsequent divergence events (starting 0.4 Ma) dividing populations located within each side of the Rift System (Fig. 3). We performed a phylogeographic analysis using Bayesian ancestral state reconstruction methods in BEAST (Fig. 4a,b); these were not conclusive, supporting a geographic origin of *C. eminii* in the Harar Massif or the SMSI, on each side of the main Rift System, with nearly equal probability. Nine migration routes were inferred by Bayesian Stochastic Search Variable Selection (BSSVS, see M&M) (Table S2.5); these seem to be arranged into parallel routes, following migration from north to south and vice versa on each side of the Rift System, and connecting also the intermediate mountain ranges (Fig. 4b).

### AFLP polymorphism, genetic diversity and structure

To complement the plastid signal (above), we carried out an analysis of fragment length polymorphism (AFLP) in the nuclear compartment. The final data set after scoring comprised 773 loci. The Bayesian software STRUCTURE assigned individuals to four geographic clusters based on patterns of genetic variance (*K* = 4, Fig. 2c; Fig. S2.2). These clusters were broadly coincident with those recovered by the haplotype network: i) a cluster of populations on the eastern side of the main Rift System (Harenna Forest, Agere Maryam and Aberdare Mts.), ii) the Abyssinian Plateau, iii) the Central Highlands, and iv) the Albertine Rift (Rwenzori Mts.). Neighbour-joining and neighbour-net diagrams (Fig. S2.3a,b) identified six well-supported groups coincident with main mountain ranges within the Rift, except for a group (Agere Maryam) that had a low bootstrap value (72.6%). The two populations of the Abyssinian Plateau were also genetically very close. Principal Component Analysis (PCO) of genetic variance also differentiated six groups: those of the eastern Rift occupied the centre of the plot, with the remaining located on the periphery (Fig. S2.4). Genetic differentiation among populations (Fst values) was higher among the western populations (Debre Markos, Mt. Elgon and Rwenzori, 0.28–0.38) than among those in the eastern side (Harenna Forest, Agere Maryam and Aberdare Mts., 0.17–0.21; Table S2.6), suggesting greater isolation among the populations west of the Rift than between those in the east. East of the Rift Valley System, a rarity index (frequency-down-weighted marker value, DW) was highest for the Harar Massif population, which also presented the highest number of polymorphic and private fingerprints (Table 2). Populations in the Central Highlands (Mt. Elgon and Cherangani Hills) had the highest DW values and a large number of polymorphic fragments, plus two fixed fragments. Populations west of the Rift Valley showed generally lower values of genetic diversity and no fixed fragments. No linear relationship was found between pairwise F_ST_ and geographic distance (Mantel analysis, *r* = 0.1884, *p* = 0.5012; Fig. S2.5). However, the correlation increased after excluding Mt. Elgon’s population from the analysis (*r* = 0.6198, *p* = 0.0560; see black stars in Supplementary Fig. S2.5). This population showed the largest genetic distance in pairwise-comparisons in relation to all other populations (Table 2.6, see Supplementary Fig. S2.5). In contrast, the population of the Abyssinian massif, which is separated by 500–1300 km from the southernmost populations (Mt. Elgon, Rwenzori), showed much lower pairwise F_ST_ values (Table S2.6). All intervals analysed with SPAGeDi (see Supplementary Fig. S2.6) gave a significant negative result, suggesting that populations are not more different with increasing distance. In agreement with this, BARRIER detected three major boundaries separating the four STRUCTURE clusters: i) Rwenzori Mountains from Mt. Elgon (100%), ii) Mt. Elgon from all areas in the east (100%), and iii) Abyssinian massif from the remaining areas (99.7%) (Fig. 2c). Hierarchical AMOVA showed the greatest genetic variance among the same four groups identified by STRUCTURE and BARRIER (Table S2.4). The relatively low among-group genetic variance (Table S2.4) might be explained by the limited number of populations sampled at each side of the Rift, and therefore should be taken with caution. Yet, the geographic structure recovered by AMOVA is fully congruent with the results from the Bayesian clustering (STRUCTURE and BARRIER) and with the Fst and DW estimates, lending support to this analysis.

## Discussion

The BEAST “nested dating” approach used here provided divergence time estimates similar to those in Mairal *et al*.^31^ for the origin of *Canarina* (8.98 My.) and the stem-age of *C. eminii* (7.14 My.) (Fig. S2.1). The first divergence event within *C. eminii* (ca. 1.92 Ma, 95% HPD 0.63–3.82 Ma, Fig. 3) postdates the age of establishment of the Afromontane forest^16^ (Early Pleistocene, ca. 2.4 Ma^32^), and is roughly coincidental with a period of aridification in East Africa, peaking at 1.7 Ma^33^,^34^. Our results also support that divergence in *C. eminii* followed the opening of the Rift Valley in the Early Pliocene, which divided the Rift System into two landstrips^35^.

A strong phylogeographic structure with the Great Rift Valley at its central axis was recovered for *C. eminii* populations. This agrees well with phylogeographic studies in other Afromontane groups (i.e, angiosperms, insects, and vertebrates, Table 1), all supporting the Rift System as an effective barrier to gene flow among populations. The depth of the phylogeographic split among STRUCTURE groups within *C. eminii* (Fig. 2c), the distribution of haplotypes (Fig. 2a,b), and the pattern of reciprocal monophyly and deep divergence times (1.92 Ma, HPD: 0.63–3.82 Ma) inferred with BEAST (Fig. 3), suggest a history of long-term isolation among populations east and west of the Rift Valley. In contrast, the large geographic distances and comparatively lower genetic distances between populations located on either side of the Rift Valley, suggest that geographic distance played a minor role in structuring patterns of genetic variation within *C. eminii*, confirmed by the lack of correlation the Mantel test establishes.

**Figure 1:**
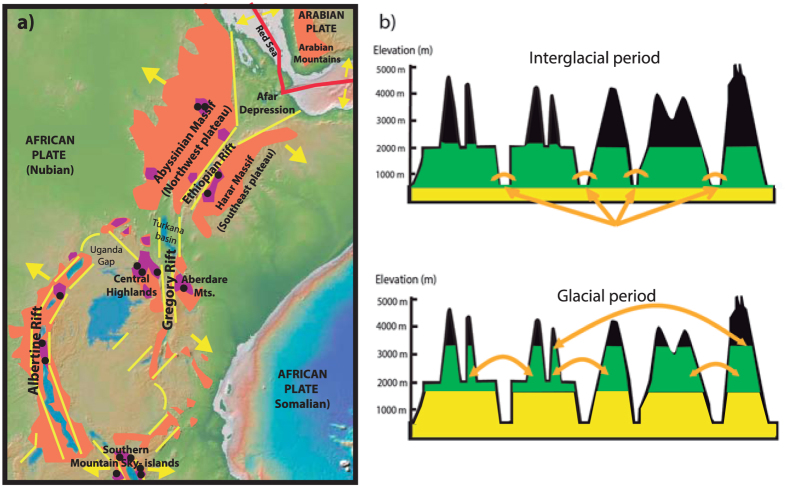
Geographic map of the Afromontane forests and main phylogeographic hypotheses. (**a**) Geographic map of Eastern Africa showing the main geographic features named in the text. The Eastern Afromontane biodiversity hotspot is shaded in orange. The distribution of *Canarina eminii* is shaded in purple. The Rift System is shown in yellow; lines represent major tectonic faults and arrows indicate the direction of relative divergent Rift movement. The red line shows the tectonic plate boundaries. The map was generated using the software GeoMapApp (v. 2.3) (http://www.geomapapp.org/)^85^. Black dots represent the sampling for *C. eminii.* (**b**) Sky-islands housing Afromontane forests (in green) separated by savannahs and open forests (in yellow), showing the two main phylogeographic hypotheses postulated to explain patterns of genetic variation in Afromontane organisms. Upper) the “mountain forest bridge” hypothesis (arrows indicate forest reconnections); Lower) the “long-distance dispersal” hypothesis (arrows indicate migration events).

Patterns of colonization and biotic assemblage in the Eastern African Mountains have been argued to follow a northern (an “African pan-temperate element”^28^,^36^,^37^) and a southern (an “Afromontane track”^38^,^39^,^40^) component. Migrants coming from the north would have needed to overcome the widest point of the Rift Valley, the Afar Triangle (Fig. 1a), which acts as a major division before reaching the Ethiopian Plateaus, while southern migrants probably needed to split into two routes when reaching the two parallel volcanic arcs of the Albertine and the Gregory Rift^39^ (Fig. 1a). Thus, dispersal into the Eastern African Mountains from the north or from the south would have been forced by topography to follow two parallel routes, one on each side of the Rift Valley^19^,^22^. This scenario is supported by the Discrete Phylogeographic Approach (DPA) analysis of *C. eminii*, showing two colonization routes that split along the topography of the Rift System (Fig. 4b). Though this method has been recently criticized as being too decisive^41^, other studies in Afromontane taxa have found similar phylogeographic connections through the east^26^,^27^,^42^ and west^8^,^43^,^44^ (Table 1).

Which mechanisms are behind this pattern of geographic isolation between the two sides of the Rift Valley for *C. eminii* and other Afromontane taxa ? As mentioned above, the origin of *C. eminii* considerably postdates the formation of the Rift, so strict vicariance is not feasible (nor for any of the groups in Table 1). However, the major faults and flooding of graben associated to the Rift activity, may have acted as a corridor filtering migration, spatially restricting dispersal to two parallel paths on either side of the Rift Valley. Indeed, the volcanic desert of Afar in Ethiopia (the hottest place on Earth), and the drier floor and deep lakes at the bottom of Rift Valley, are ecologically very different from the surrounding higher elevation areas^45^,^46^, and constitute ecological barriers to dispersal (i.e., hostile environments that are beyond the tolerance limits of montane organisms). It would be interesting to test this hypothesis using genetic and ecological evidence in other lineages exhibiting a similar pattern of genetic isolation along the Rift Valley (Table 1).

**Figure 2:**
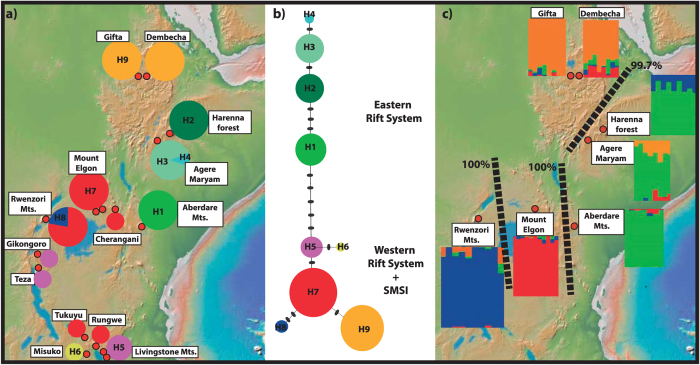
Plastid and nuclear datasets analysed for *Canarina eminii*. (**a**) Haplotype distribution. Red dots represent the geographic location of populations and pie charts show the frequency of occurrence of each haplotype. (**b**) Statistical Parsimony network inferred using the DNA plastid sequences by TCS. Black dashes on long connecting lines indicate nucleotide changes. Circle size is proportional to the frequency of haplotypes. Each haplotype is shown in a different colour, where codes (H1 to H9) correspond to the haplotypes shown in Fig. 2a. (**c**) Phylogroups using AFLP markers. Histograms showing the Bayesian clustering of individuals within populations (STRUCTURE); colours represent the proportion of individual membership to each inferred Bayesian group. Dashed lines indicate barriers to gene flow and their percentage, as inferred by BARRIER. The map was generated using the software GeoMapApp (v. 2.3) (http://www.geomapapp.org/)^85^.

Eastern Africa has experienced several climate fluctuations over the last 800,000 years, resulting in mountain forests extending and retracting successively, with further isolation and bottlenecking events for some species^47^,^48^. The Afromontane forest probably did not descend far enough to reach the bottom of the Rift Valley graben^25^ during the humid-climate expansion periods, and a widespread montane forest only seems likely on the Ethiopian plateaus, on either side of the Rift Valley^25^. However, some species might have had opportunities for gradual SSD migration through grasslands and open forest corridors between mountains^25^. This would have resulted in sister phylogroups occupying separate mountain ranges on either side of the Rift Valley, which could have been followed by *in situ* diversification during the arid glacial periods^25^,^49^. Patterns of genetic variation in *C. eminii* agree with this hypothesis, suggesting long-term isolation of populations in mountain enclaves on each side of the African Rift. High genetic differentiation and exclusiveness (Fig. 2a–c, Table S2.4, see Supplementary Fig. S2.3 and S2.4) was observed among populations on each sky-island, in agreement with other Afromontane phylogeographic studies^8^,^50^,^51^,^52^ and those in the Ericaceous^42^ and Afroalpine^26^,^27^,^53^ vegetation belts. In contrast to the glaciation-induced latitudinal range shifts in European taxa, which apparently resulted in a loss of genetic diversity (i.e., extinction and bottlenecks^54^), repeated events of fragmentation and reconnection during Pleistocene glacial/interglacial cycles may have contributed to the accumulation and maintenance of genetic diversity in the Eastern African mountains, and could explain their current status as centres of endemicity^52^,^53^. This agrees well with the hypothesis of a more moderate effect of glacial cycles at equatorial latitudes, in contrast with regions at higher latitudes^54^.

**Figure 3:**
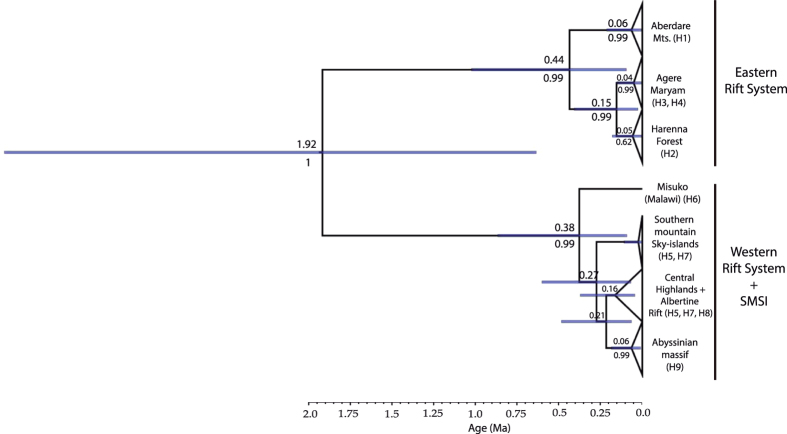
Maximum clade credibility (MCC) tree obtained from the BEAST analysis of pDNA haplotypes of *Canarina eminii*. Blue bars show 95% HPD credibility intervals. Numbers above branches show mean ages and numbers below branches indicate Bayesian posterior clade support values.

Another explanation for the east/west connectivity pattern and the observed low genetic variance between populations on each side of the Rift in *C. eminii* and other Afromontane endemics, is long-distance dispersal of seed and pollen through vectors such as wind, insects, or birds; this in turn could have been reinforced by wider forest coverage in the past, i.e., greater area availability for seedling establishment^25^. Long-range seed dispersal is likely the best explanation for connections among populations of *C. eminii* across the Turkana Basin and the Uganda Gap, which probably constitute ecological barriers (climatically unfavourable land) for gradual SSD migration through forest connections. Results from the plastid DNA suggest seed dispersal on each side of the Rift Valley, with more recent connectivity among the eastern side populations (Figs 2a and 3). On the other hand, the strong interpopulation structure found in the nuclear AFLP data suggests limited pollen dispersal (Fig. 2c). This could be explained by the strong territoriality and reduced mobility of Nectariniidae birds, the presumed pollinators of *C. eminii*. Some recent studies on these birds have revealed cryptic species within the same lineage occupying different mountain ranges, probably due to low connectivity between populations^44^,^55^. This pattern might have later been reinforced by a landscape strongly transformed by agriculture.

**Figure 4:**
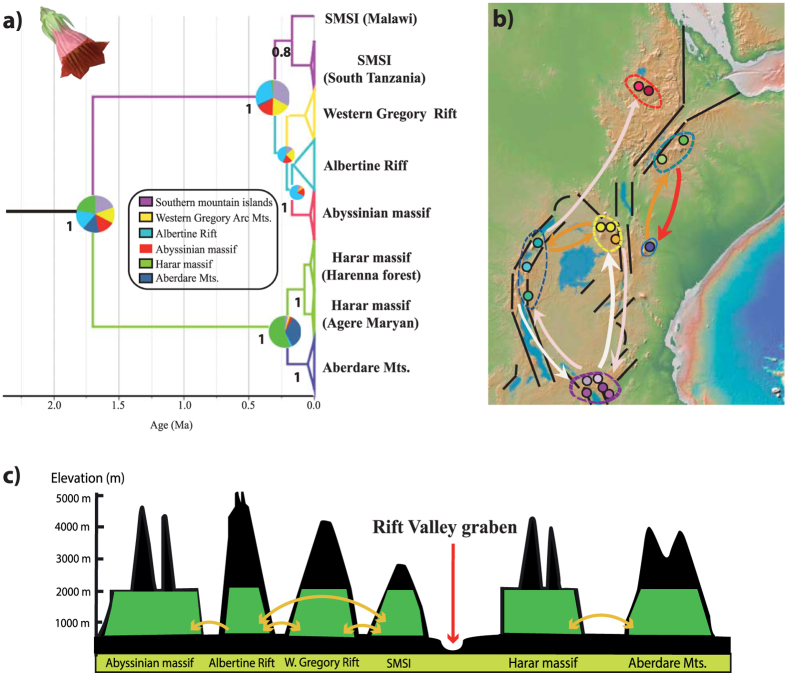
Phylogeographic analysis and reconstruction of the colonization of *Canarina eminii*. (**a**) BEAST MCC tree showing the Bayesian ancestral range reconstruction analysis^63^. Coloured branches (see legend) represent the ancestral range with the highest posterior probability for each population; node pie charts show marginal probabilities for alternative ancestral ranges. Numbers below branches represent Bayesian posterior probabilities. (**b**) Map representing migration events that receive a BF support > 3, as recovered by BSSVS; colour tint is proportional to the support (dark red > orange > pale pink > white). The map was generated using the software GeoMapApp (v. 2.3) (http://www.geomapapp.org/)^85^ (**c**) A hypothetical reconstruction of the colonization of the Afromontane forest by *Canarina eminii* during interglacial periods based on our data and literature of other Afromontane groups. Orange arrows show possible short to medium distance dispersal events between isolated mountain ranges or across forest galleries. Expansion of the Afromontane forest (in green) during interglacial periods may have facilitated migration across Stepping Stone dispersal or forest bridges.

Both plastid and nuclear genetic data suggest a pattern of greater historical isolation among populations located in the western side of the Rift Valley than among those in the east (Fig. 2, Table 2). Whether this is associated to greater past connectivity through forest patches (mountain-bridge hypothesis) in the east, or whether it reflects a more recent historical pattern associated to agricultural activity, is not clear. Similar to other studies^8^,^50^,^51^, the highest level of genetic diversity was found in the Harar Massif, an area known for harbouring the best-preserved fragments of Afromontane forest (Table 2: Agere Maryam and Harenna Forest). This area may have acted as an important refuge for Afromontane forest organisms during glacial maxima. In the DPA analysis of *C. eminii*, the Harar Massif was inferred as the source of migration events on the eastern side of the Rift (Fig. 4b). For the western Rift, the SMSI was suggested as the ancestral area: these mountains harbour the highest plastid genetic diversity (Table 2), though these results could not be corroborated with the nuclear data (no AFLP sampling). The two arcs of the Rift Valley (Albertine and Gregory Rift) meet geographically in the north on the Central Highlands (Mt. Elgon and Cherangani Hills) and in the South in the SMSI (Fig. 1). These two regions (Mt. Elgon- Cherangani and the SMSI) apparently act as secondary contact points where the two parallel migration routes mentioned above interconnect^19^,^44^, and have been described as cradles of new genetic diversity^53^. This is congruent with our results in *C. eminii*, showing Mt. Elgon and the SMSI as a crossroad linking both sides of the west Rift System in the DPA analysis (Fig. 4), and both exhibiting high levels of genetic diversity (Table 2). Mount Elgon is also the oldest and most species-rich volcano of Eastern Africa^13^,^56^, with endemic lineages that are related to groups in either the eastern^43^ or the western side of the Rift Valley^8^,^43^. In contrast, the Afar Depression and the arid zone around the Turkana Basin seem to have acted as major barriers against gene flow (Fig. 1a and 4b). The Afar Depression separates the eastern and western Ethiopian populations, north of the two volcanic arcs, while the aridity around the Turkana Basin and the Uganda Gap, might have constituted an important obstacle to gene flow between the eastern and western populations^57^. The low haplotype diversity found in the Abyssinian massif population could reflect long-term isolation due to the influence of glaciations, though a low DW value suggests that recent dispersal could be a more likely explanation.

In summary, our study suggests that the topography and land formations of the Great Rift Valley underlie the phylogeographic pattern of east/west vicariance observed in *C. eminii* and other Afromontane endemic species. Though population sampling was limited to discriminate between the “mountain-forest” bridge and the LDD hypotheses, Pleistocene climatic oscillations and cyclical expansion and contraction of the Afromontane forest appear to have played a role in structuring levels of genetic variation in these species. Short-range dispersal among forest patches on each side of the Rift probably was complemented by LDD across some regions such as the Turkana Basin or the Uganda Gap, historically devoid of montane forests. Isolated sky-islands such as Mount Elgon or the Harar Massif acted as both refuges and cradles of genetic diversity. Given that the forested areas of Eastern Africa are currently in serious decline^9^ —especially the northern Ethiopian highlands subject to extensive logging— and that many of these harbour high levels of genetic variability (see Table 1, our study), designing new measures for the protection of these regions (e.g., new natural parks) may be crucial to preserve the endemic and highly-threatened Afromontane forest biota.

## Methods

### Sampling and DNA sequencing

The geographic distribution of *C. eminii* extends from the Ethiopian massifs to southern Tanganyika^28^. Despite our sampling effort (two field expeditions in 2009, 2015 to Ethiopia and one in 2010 to Kenya-Uganda), we failed to find *C. eminii* in many of the localities recorded by Hedberg in his classic monography of this species^28^. Much of the forest in these localities is highly degraded, threatened by the advancement of extensive agriculture, cattle rising, forestry, and other human activities (see Table S2.1 for a list of the visited localities for this study and a detailed description of the sampling). Despite this, we were able to sample fresh plant tissue from seven populations of *C. eminii* (Table S2.2): two populations in the Abyssinian massif (Gifta and Dembecha), two in the Harar Massif (Harenna forest and Agere Maryam), one in Rwenzori Mts. (Albertine Rift), one in Mt. Elgon (west of the Gregory Rift), and one in Aberdare Mts. (east of the Gregory Rift) (Fig. 2). Whenever possible leaves from 8–17 plants, 7–15 m apart were collected from each population. In addition, we added nine localities, obtained from old herbarium specimens (Royal Botanic Gardens, Kew): Cherangani (Kenya), Sasa trail in Mt. Elgon (Uganda), Gikongoro (Rwanda), Teza (Burundi), Tukuyu, Mwakelele, Livingstone Mountains, Mporoto Mountains (Tanzania) and Misuku (Malawi). Altogether, we sampled 16 localities of *C. eminii* (Table S2.2), representing all major geographic blocks in the distribution of the species (Fig. 1a). Three plastid (pDNA) regions were sequenced for a total of 79 individuals. For details on PCR amplification and sequence alignment see Appendix S1.1 and Table S2.3 in the Supplementary Material. Sources of the material examined, location of vouchers, GenBank accession numbers, and full references are listed in Table S2.2.

### Phylogeographic analysis

Haplotype analyses were done on a concatenated dataset comprising all three sequenced plastid regions (see above). Genealogical relationships among haplotypes were inferred via the statistical parsimony algorithm^58^ implemented in TCS 1.2.1^59^. The number of mutational steps resulting from single substitutions among haplotypes was calculated with 95% confidence limits, with gaps coded as missing data. Summary statistics for genetic diversity calculated for each population were: number of haplotypes H (n), haplotype diversity (Hd), and nucleotide diversity (π); using DnaSP 5.1 with the option “not consider gaps” selected^60^.

Haplotype divergence times were estimated using Bayesian relaxed clocks implemented in the software package BEAST v.1.7.5^61^ and using the ‘nested dating approach’ described in Mairal *et al*.^31^; in this approach, a high phylogenetic-level dataset, including representatives of all three species of *Canarina* and nine outgroup taxa from Platycodoneae and Campanulaceae, was used to inform the clock rate of a linked population-level dataset of *C. eminii*, under a mixed Yule-coalescent model^62^. Congruence among results from the three plastid markers was tested by comparing clade support values for individual clades. See Appendix S1.2 for more details on these analyses.

The Discrete Phylogeographic Approach (DPA) of Lemey *et al*.^63^, implemented in BEAST, was used to infer ancestral ranges and to trace the history of migration events in *C. eminii*. It is based on a continuous-time Markov Chain process where the discrete states correspond to the geographic locations and the state transition rates to the migration rates between areas (Ronquist & Sanmartín, 2011). Bayesian Stochastic Search Variable Selection (BSSVS, Lemey *et al*.^63^) was used to identify the rates (colonization routes) that are best supported by the data, using a cut off value of three for the Bayes Factor comparison. We defined six discrete areas, assigning each plateau and Rift mountain ranges to a different area^15^,^31^: 1) Harar massif, 2) Abyssinian massif, 3) East Gregory Rift (Aberdare Mountains), 4) Central Highlands (Mount Elgon and Cherangani Hills), 5) Albertine Rift (Rwenzori, Rwanda and Burundi), and 6) Southern Mountain Sky-Islands (SMSI: South Tanzania and Malawi) (see Appendix S1.3 for more details).

### AFLP fingerprinting

Laboratory molecular protocols for the AFLP analysis^64^ were implemented using the AFLP plant mapping kit (Applied Biosystems). We could not use the individuals sampled from herbarium collections for AFLP fingerprinting, as this approach is quite sensitive to the quality of the starting DNA and thus requires well-preserved material^65^. Therefore, sampling of populations in the nuclear dataset was more reduced than in the pDNA sequence dataset. Genomic DNA was digested with the enzymes *Eco*RI and *Mse*I and linked to the adaptors *Eco*RI (5’-CTCGTAGACTGCGTACC-3’/5’-AATTGGTACGCAGTCTAC-3’) and *Mse*I (5’- GACGATGAGTCCTGAC-3’/5’-ATCTCAGGACTCAT-3’). We tested 32 combinations of selective primers and chose the four pairs that produced the most polymorphic and clear profiles: 1-*Eco*RI_6-FAM_-ACT/*Mse*I-CAA; 2-*Eco*RI_6-FAM_-ACT/*Mse*I-CAT; 3-*Eco*RI_VIC_-AGG/*Mse*I-CTA, and 4-*Eco*RI_VIC_-AGG/*Mse*I-CTT.

Amplified fragments were analysed using GeneMapper 3.7 software (Applied Biosystems), and peaks ranging between 100 and 500 bp recorded. AFLP Scorer software^66^ was employed to run a reproducibility test, with the maximum acceptable error for each primer combination fixed at < 5%^67^. The AFLPdat R package^68^ was used to determine the numbers of private fragments per population. Only unambiguous fragments shared among duplicates were scored. Data reliability was assessed by comparison of duplicates, using one or two individuals per population (21 tests). The reproducibility value was 88–100%, with a mean of 97.5%.

### AFLP data analysis

The resulting AFLP presence/absence matrix was analysed using AFLPSURV v.1.0^69^ to estimate Nei’s gene diversity (Hj), pairwise differentiation among subpopulations (F_ST_), the percentage of polymorphic fragments per population (P), and the bootstrapped Nei’s genetic distance matrix between individuals and populations^70^,^71^. The inbreeding coefficient (F_IS_) was set to 0.1 as suggested by Hardy^72^. The permutation test involved 20,000 permutations. In addition, a Bayesian method was employed to estimate allelic frequencies, using a non-uniform prior distribution^73^. Ten thousand permutations were performed to calculate F_ST_ values. Genetic distances between individuals, populations and geographic groups were also calculated. We used AFLPdat^74^ to calculate DW value per population, equivalent to the weighted endemism value^75^,^76^; this value is expected to be high in long-term isolated populations where rare markers should accumulate due to mutations, whereas newly established populations are expected to exhibit low values, thus helping to differentiate recent dispersal from more ancient isolation.

To distinguish genetic groups of individuals in the AFLP dataset, a comparison was made by constructing a pairwise similarity matrix for all individuals: Dice’s coefficient was calculated and the resulting matrix was transformed using principal coordinates analysis (PCO) with Ntsys v.2.1^77^. Neighbour-nets of AFLP data were also calculated, both for individuals and populations, using the SplitsTree v.4.10 software^78^. To quantify the amount of genetic differentiation attributable to geographic and population subdivision, a hierarchical analysis of molecular variance was performed^79^ in ARLEQUIN v.3.0 (Table S2.4). For assessing the structure of populations, we used the Bayesian method implemented in STRUCTURE 2.2^80^,^81^, assuming admixture conditions and uncorrelated allele frequencies between groups, 500,000 generations (plus a burn-in of 100,000) were run for *K* values of 1–10, with ten repetitions each. For each *K* value, only the run with the highest maximum likelihood value was considered. The LnP (D) for the successive decomposition of groups was used in all STRUCTURE analyses^82^. To test the effect of the spatial distance on the genetic structure of the *C. eminii* populations, correlations between genetic (measured as F_ST_) and spatial distances between pairs of populations were determined using the Mantel permutation procedure in Ntsys. The genetic distance matrix used was based on the presence/absence matrix; the geographic distance matrix was based on absolute distances between the geographic coordinates for each collected population. In addition, the kinship multilocus coefficient (F_IJ_) was estimated using SPAGeDi^83^ to determine the spatial structure of the examined populations, taking spatial distances into account in the analyses. BARRIER v.2.2^84^ was used to identify possible geographic locations acting as major genetic barriers among *C. eminii* populations, based on genetic distances. The significance of these was examined with 1000 bootstrapped distance matrices obtained using AFLPsurv.

### Data Accessibility

DNA sequences: Genbank Accession nos KF028817–KM189329. GenBank accessions, sampling locations and/or online-only appendices uploaded as online Supplemental material.

## Additional Information

**How to cite this article:** Mairal, M. *et al*. Geographic barriers and Pleistocene climate change shaped patterns of genetic variation in the Eastern Afromontane biodiversity hotspot. *Sci. Rep.* **7**, 45749; doi: 10.1038/srep45749 (2017).

**Publisher's note:** Springer Nature remains neutral with regard to jurisdictional claims in published maps and institutional affiliations.

## Supplementary Material

Supplementary Information

## Figures and Tables

**Table 1:** Phylogeographic studies of organisms showing genetic variation structured around the Rift System.

*Organism*	Phylogeographic disjunctions in the Ethiopian Rift	Phylogeographic disjunctions in the Volcanic arcs	Method (Reference)
***Angiosperms*****(habitat)**			
*Coffea arabica* (Afromontane)	Two distinct groups across the Ethiopian Rift	—	ISSR; microsatellites^50^,^86^
*Lobelia giberroa* (Afromontane)	Two distinct groups across the Ethiopian Rift: 1. Simien- Choke; 2. Chilallo-Bale-Gara Muleta	Two distinct groups across the Gregory Rift	AFLP^8^
*Hagenia abyssinica* (Afromontane)	Structure both sides of the Rift with rare long-distance dispersal events crossing the Rift	—	microsatellites^51^
*Cordia africana* (Afromontane)	Three different groups across the Ethiopian Rift: 1. Northwest plateau; 2. South-west Ethiopia; 3. Southeast plateau	—	AFLP and microsatellites^87^
*Juniperus procera* (Afromontane)	Two relatively different groups across the Ethiopian Rift: 1.Goba-Yabelo; 2. Chilimo-Suba-Ziquala-Washa	—	AFLP^88^
*Prunus africana* (Afromontane)	—	Two distinct groups across the Uganda Gap: 1. Albertine Rift and western Gregory Rift; 2. Eastern Gregory Rift	Plastid haplotypes, SSR^52^,^89^
*Warburgia ugandensis* (Afro- montane transitional forest)	—	Two distinct groups across the Uganda Gap: 1. Albertine Rift and western Gregory Rift; 2. Eastern Gregory Rift	AFLP^90^
*Erica trimera* (Ericaceous)	No difference for AFLP, but different haplotypes	A complex pattern in the Gregory Rift	AFLP and plastid haplotypes^42^
*Arabis alpina* (Afroalpine)	Two distinct groups across the Ethiopian Rift: 1. Simien, Gara M.; 2. Choke, Elgon, Meru, Kilimanjaro	A complex pattern in the Gregory Rift	Plastid haplotypes^19^
*Cardus schimperi* (Afroalpine)	Two distinct groups across the Ethiopian Rift: 1.Simien; 2. Elgon-Aberdare-Bale	Mt. Kenya population is a different subspecies; while populations from Mt. Elgon and Aberdare are closely related	AFLP^26^
*Trifolium cryptopodium* (Afroalpine)	Two distinct groups across the Ethiopian Rift: 1. Simien Choke; 2. Bale-Aberdare–Elgon-Kilimanjaro	Populations related across the Rift Valley	AFLP^26^
*Deschampsia cespitosa* (Afroalpine)	—	Two distinct groups across the Rift Valley: 1. Rwenzori; 2. Kilimanjaro-Bale	AFLP^27^
*Senegalia senegal* (savannah, semi-desert)	—	Two distinct groups across the Gregory Rift with mixed population (Marigat): 1. Western side; 2. Eastern side.	microsatellite^91^
*Senegalia mellifera* (savannah)	—	Two distinct groups across the Gregory Rift: 1. Western side; 2. Eastern side; 3. Central Gregory Rift	microsatellite^49^
***Vertebrates***			
Ostrich *Struthio camelus*	Two subspecies both sides of the Rift (*molybdophanes*/*camelus*)	Two subspecies both sides of the Rift (*Molybdophanes*/*camelus*)	mtDNA^46^
Olive sunbird *Nectarinia*	—	Two distinct species across the Rift Valleys. 1. Western Rift Valley *Nectarinia obscura*. 2. Eastern Rift Valley *Nectarinia olivacea*	mtDNA^44^
Springhare *Pedetes capensis*	—	Two distinct groups across the Rift Valleys: eastern populations/southern populations	mtDNA^92^
Wildebeest *Connochaetes taurinus*	—	Two subspecies both sides of the Gregory Rift. 1. Western Gregory Rift: sbsp. *mearnsi* (Loliondo, Masai-Mara) 2. Eastern Gregory Rift: sbsp. *albojubatus* (Nairobi and Amboseli)	mtDNA^93^
African wild dog *Lycaon pictus*	—	Two distinct groups across the Gregory Rift. 1. Eastern clade. 2. Southern clade	mtDNA and microsatellites^94^
Sable antelope *Hippotragus niger*	—	Two distinct groups across the Gregory Rift: 1. West Tanzania and Kenya; 2. East Tanzania)	mitochondrial DNA and cyt. *b*^95^
Ethiopian wolf *Canis simensis*	Three distinct groups across the Ethiopian Rift: Northwest plateau (1. Wollo/Shoa; 2. Simien/Mt. Guna) and southeast plateau (3. Arsi/Bale)	—	mtDNA^22^
Lion *Panthera leo*	—	Two distinct groups across the Gregory Rift: 1. eastern (Tsavo-Transvaal); 2. western (Aberdare)	cytochrome *b* and NADH desh. subunit 5^96^
Baboon *Theropithecus gelada*	Two distinct groups across the Ethiopian Rift (northwest and southeast plateau)	—	RFLPs^97^
Grass mouse *Lemniscomys striatus*	—	Two distinct groups across the Rift Valleys: Gregory Rift/Albertine Rift	cytochrome *b*^98^
Rodent *Mastomys natalensis*	—	Two distinct groups across the Rift Valleys: eastern populations/southern populations	cytochrome *b*^99^
African clawed frogs (*Xenopus clivii* and *X. largeni*)	Two distinct groups across the Ethiopian Rift (northwest and southeast plateau)	—	mtDNA, autosomal loci^100^
Ethiopian anurans (*Tomopterna, Amietia, Leptopelis, Ptychadena*)	Two distinct groups across the Ethiopian Rift	—	Several mitochondrial and nuclear genes (101)
***Insects***			
Mosquito *Anopheles gambiae*	—	Two distinct groups across the Rift Valleys: eastern populations (Kimili, Asembo bay, Kisian, Awendo)/western populations (Malindi, Jego)	microsatellites^102^,^103^,^104^,^105^
Tsetse fly *Glossina pallidipes*	—	Two distinct groups across the Rift Valley: East Rift Valley (Dakabuko, Alangoshira, Shimba Hills, Kibwezi); West Rift Valley (Nguruman, Shompole, Marech)	Allozimes, microsatellites, mitochondrial loci^106^
Mosquito *Anopheles funestus*	—	Two distinct groups across the Rift Valley: western Kenya pop. (Mbita, Udhoro)/Coastal populations (Majajani, Magaoni)	microsatellites^107^
Termite *Scledorhinotermes lamanianus*	—	Two distinct groups across the Rift Valley: eastern populations/western populations	AFLPs^57^

**Table 2:** Descriptors of within-population genetic diversity in the cpDNA haplotypes and AFLP markers for each population studied of *Canarina eminii*.

Haplotypes							AFLPs					
Population	Mountain range	N° samples	Haplotypes	H (n)	H (d)	π	N° samples	N° of polymorphic fragments (% in brackets)	Hj (se)	N° of private fragments (% in brackets)	N° of fixed private fragments	DW index
**West of the Rift**												
Gifta, Debre Markos (Ethiopia)	Abyssinian massif (Northwest Plateau)	8	H9	1	0	0	8	477 (61.7)	0.244 (0.0071)	8 (1.68)	0	93,187
Dembecha, Debre Markos (Ethiopia)	Abyssinian massif (Northwest Plateau)	7	H9	1	0	0	8	443 (57.3)	0.208 (0.0069)	6 (1.35)	0	99,213
Rwenzori Mts. (Uganda)	Albertine Rift	12	H7, H8	2	0.303	0,00026	10	417 (53.9)	0.193 (0.007)	6 (1.439)	0	111,177
Gikongoro-Teza (Rwanda-Burundi)	Albertine Rift	2	H5	1	0	0	–	—	—	—	—	—
**Central highlands**												
Mt. Elgon 1 & 2-Cherangani Hills (Kenya)	Central sky-islands	19	H7	1	0	0	11	477 (61.7)	0.183 (0.0063)	5 (1.048)	2	140,694
**East of the Rift**												
Agere Maryam, Yirga (Ethiopia)	Harar massif (Southeast Plateau)	9	H3, H4	2	0.222	0.0001	8	495 (64.0)	0.241 (0.0067)	13 (2.626)	1	108,327
Harenna Forest, (Ethiopia)	Harar massif (Southeast Plateau)	8	H2	1	0	0	9	491 (63.5)	0.224 (0.0067)	10 (2.037)	2	128,342
Aberdare Mts. (Kenya)	Aberdare Mts. (sky-island)	9	H1	1	0	0	7	435 (56.3)	0.208 (0.0067)	8 (1.839)	2	91,057
**Southern Mountain Sky-Islands**												
Misuku-Tukuyu-Rungwe-Livingstone Mts. (Tanzania-Malawi)	Southern Mountain Sky-islands	5	H5, H6, H7	3	0,4	0,00032	—	—	—	—	—	—

Abbreviations: H(n): number of haplotypes; H(d): haplotype diversity; π: nucleotide diversity; Hj (se): Nei’s gene diversity (standard error); DW: frequency-down-weighted value.
