# Supplementary material for: Geographic barriers and Pleistocene climate change shaped patterns of genetic variation in the Eastern Afromontane biodiversity hotspot
Source: Sci Rep. 2017 Apr 11;7:45749. doi: 10.1038/srep45749 (PMC5387718; doi:10.1038/srep45749)
Supplement: Supplementary Information [file srep45749-s1.pdf]

SUPPLEMENTARY INFORMATION

Article title

Geographic barriers and Pleistocene climate change shaped patterns of genetic variation in the Eastern Afromontane biodiversity hotspot.

Authors

Mario Mairal[*1], Isabel Sanmartín[1], Alberto Herrero[1], Lisa Pokorny[2], Pablo Vargas[1], Juan J. Aldasoro[†3], and Marisa Alarcón[†3]

[1] *Real Jardín Botánico (RJB-CSIC), 28014 Madrid, Spain*

[2] *Royal Botanic Gardens, Kew (RBGK), Richmond, Surrey, TW9 3DS, UK*

[3] *Instituto Botánico de Barcelona (IBB-CSIC-ICUB), 08038 Barcelona, Spain*

SUPPORTING INFORMATION

Appendix S1. Expanded material and Methods

Appendix S2. Supplemental Tables and Figures

Appendix S3. Supplemental references

Appendix S1 Expanded Material and Methods

S1.1 DNA amplification, sequencing and alignment DNA

DNA of 72 individuals was extracted using the DNeasy Plant Mini Kit (QIAGEN Inc., California, USA) according to the manufacturer's instructions, from 18-23 mg of silica-gel dried leaves obtained from the fresh plant tissue collected from the field expeditions. PCR amplifications were performed using an Eppendorf Mastercycler Epgradient S (Westbury, NY). Table S3.3 shows the primer sequences for each region. The three intergenic spacers were amplified using the following PCR settings: 1) for *rpl*32-*trn*L_{UAG} : 1 min pretreatment at 95°C, 30 cycles of 1 min at 96°C, 1 min at 52°C, and 2 min at 72°C; 2) for *trn*S_{GCU}-*trn*$_{GUCC}$: 1 min pre-treatment at 96°C, 30 cycles of 45 s at 96°C, 1 min at 54°C, and 1 min 30s at 72°C; and 3) for *pet*B1365–*pet*D738: 1 min pre-treatment at 96°C, 30 cycles of 1 min at 95°C, 2 min at 49°C, and 2 min at 72°C. All these reactions ended with a final extension step for 10 min at 72°C. Amplified products were treated with ExoSAP-IT (USB Corporation, Ohio) and submitted to Macrogen Inc. (Seoul, South Korea) for sequencing. We had difficulties to amplify some of the herbarium specimens, for this we developed internal primers and performed nested-PCRs. The resulting sequence data were assembled and edited using SeqEd v. 1.0.3 software (Applied Biosystems, California, USA). Sequences for each region were aligned using MAFFT software (Katoh *et al.*, 2005). Sequences were manually adjusted where necessary by following alignment rules described in Kelchner (2000), and checked through the use of Bioedit software (Hall, 1999).

S1.2 Divergence time analyses

Model-fit of nucleotide substitution models was assessed via the Bayesian Information Criterion (BIC) as implemented in jModelTest 0.1.1 (Posada, 2008). The higher-level data set was calibrated with fossil-derived secondary age estimates (see Mairal *et al.* 2015), while the tree prior was unlinked to apply a coalescent constant size model to the population-level data set and a stochastic birth–death (Yule) prior to the species-level one (Mairal *et al.* 2015). The clock model was set to an uncorrelated log-normal prior to accommodate the change in mutation rate from species to populations, with a uniform distribution for the ucld.mean (10^{-4}– 10^{-1}) and a default exponential distribution for the ucld.stdev; the substitution model was set to GTR+G. Two MCMC chains were run for 50 million generations, sampling parameters every 1000 generation. We used Tracer v1.6 (Rambaut *et al.* 2007) to verify the following: whether a stationary distribution was attained, whether there was convergence among chains and whether effective sample sizes (ESS values) were >200 for all parameters. A 10% burn-in of the sampled populations was discarded (5 million). Post-burn-in trees were summarized into a maximum clade credibility tree using TREEANNOTATOR v.1.6.1, with mean values and 95% credible intervals for nodal ages, and were visualized in FIGTREE 1.3.1 (Rambaut & Drummond 2009).

S1.3 Bayesian discrete phylogeographic analyses

This method implements a continuous-time Markov Chain (CTMC) process to model biogeographic evolution, in which the discrete states correspond to geographic locations of the sequences and transition rates between states to migration rates or dispersal between areas (Ronquist & Sanmartín, 2011). Migration rates between areas and the geodispersal rate scaler were modelled using default gamma prior distributions

(Lemey *et al.*, 2009). Two replicate searches of 20 million generations each, sampling every 1000th generation, were combined in TreeAnnotator, after removing the 10% burn-in, to produce a maximum clade credibility (MCC) tree. Bayesian stochastic search variable selection (BSSVS, Lemey *et al.*, 2009) was used to identify the rates (colonization routes) that are best supported by the data.

Appendix S2 Supplemental Tables and Figures.

Table S2.1. List of the visited localities for this study and description of the sampling.

**Country**	**Hedberg collections**	***C. eminii* collections (X: explored, but not found)**	**Commentaries (not explored not explored)**
**Ethiopia**	Damot near source of Abay river	-	deforested
	Burye, road to Lake Tana	Dembecha (north of Debre Marcos), A9979	deforested, a few scattered trees contain plants of *C. eminii*
		Gifta (north of Debre Marcos), A9982	
	Shoa: Kachise (near Kachissy)	x	deforested
	Shoa, Entotto	x	deforested
	Shoa: Addis Ababa	x	deforested
	Shoa: Little Akaki river	x	deforested
	Shoa: Mannaghescia (Mannagascià)	x	deforested
	Shoa: Holeta (Oletta), valle di Metcha Coriccia	x	deforested
	Borana people, N of Agere Mariam	Agere Maryan, Yirga Chefé Road, A10060	*C. eminii* abundant
	Bale	Harenna Forest, Negele to Goba road, A10317 and A10322	*C. eminii* abundant
	Gallanot exploredSidamo: Welega (Wallega), Kidami	not explored	not explored
	Gallanot explored Sidamo: Shisha, Lake Uombo (Wombo)	not explored	not explored
	Gallanot explored Sidamo: Cencia	not explored	not explored
**Uganda**	Ruwenzori, Kangasaba	x	sparsely forested
	Ruwenzori, Mahoma Valley near Nyabitabu (Nyakalengija)	Ruwenzori Mts, Nyabitaba track, Sánchez Meseguer & Aldasoro 18	*C. eminii* abundant
	Mt. Elgon, W. slope between Bulambuli y Butandiga	Mt. Elgon, Sánchez Meseguer & Aldasoro 69	*C. eminii* abundant
	Mt. Elgon, Buginyanya	Mt. Elgon, Sánchez Meseguer & Aldasoro 71	*C. eminii* present
	N of Mt. Elgon at Kyessweri R. Sipi and Kabururon R. Sundet (Sebei)	Mt. Elgon, Sánchez Meseguer & Aldasoro 75	*C. eminii* present
	Mbale dist: Mt Elgon, Bumoni	not explored	not explored
	Bugisu distr., Bulago	not explored	not explored
	Karamoja Distr: Rom Mts	not explored	not explored, entry forbidden
	Karamoja Distr: Langia Mts	not explored	not explored, entry forbidden

	Karamoja Distr: Monte Kadam	not explored	not explored, entry forbidden
	Karamoja Distr: Monte Moroto	not explored	not explored, entry forbidden
**South Sudan**	Imatong Mts.	not explored	not explored, entry forbidden
	Lomwaga Mts.	not explored	not explored, entry forbidden
**Kenya**	Kapolet (Kaboret) forest	not explored	not explored
	Cherangani Hills	x	sparsely forested
	Elgeyo Escarpment	x	sparsely forested
	Kapsabet, Yala river	x	sparsely forested
	Nyanza Province	x	sparsely forested
	Aberdare Range	Aberdare Mts, Sánchez Meseguer & Aldasoro 103	*C. eminii* abundant
	Aberdare (east), Tuso	x	sparsely forested
	Dimbilil river, SW Mau	not explored	not explored
	Mt. Kenya, Embu distr., Thiba camp	x	sparsely forested
	Mt. Kenya, Kirinyaga distr.	x	sparsely forested

Despite intensive sampling effort (two field expeditions in 2009, 2015 to Ethiopia and one in 2010 to Kenya-Uganda), we failed to find *C. eminii* in many of the historically recorded localities (Hedberg, 1961). During the present field work, the number of localities where *C. eminii* grow in the northern Ethiopian Plateaus, and the Elgon Mt. was much smaller than that reported by Hedberg (1961). The highlands of northern Ethiopia as well as many parts of Kenya, now given over to cultivation or pasture, are largely devoid of forest vegetation. We noted that much of the forests in these localities were highly degraded, having either completely disappeared or being threatened by the advancement of extensive agriculture, cattle rising, forestry and other human activities. Although that species appeared there in previous studies, currently the absence of forested patches and adequate phorophytes likely impeded this species to survive there. All these difficulties hampered a deeper sampling of *C. eminii*. Moreover, some of the localities could not be visited due to security concerns, such as: South Sudan, eastern Congo, Burundi, South Sudan, and eastern Congo borders were not

allowed to be visited during our sampling period. Besides, there are some places in north of Uganda where the authorities prohibit any entry.

Table S2.2. Plastid sequences of *C. eminii* newly generated for this study: localities and geographical coordinates, sample collector, and GenBank accessions number. Voucher specimens of all sampled species and populations were deposited in the herbarium of the *Instituto Botánico de Barcelona*- CSIC (BC).

**Voucher specimen** *Canarina eminii* Asch. ex Schweinf.	**trnS–trnG accession number**		***pet*B–*pet*D accession number**		***rpl*32-*trn*L^{UAG} accession number**
Ethiopia, Gifta, Debre Markos to Bahir Da Road, N of Bure, 09/08/2009, 2478m, 10º 50' 29" N, 37º 02' 56" E, *Aldasoro & Alarcón 9982* (BC)	KF028817	KM189335	KF028856	KM189234	KM189267
	KF028818	KM189336	KF028857	KM189235	KM189268
	KF028819	KM189337	KF028858	KM189236	KM189269
	KM189334	KM189338	KM189233	KM189237	KM189270
Ethiopia, between Dembecha and Bure, Debre Markos to Bahir Dar Road, 08/08/2009, 2493m, 10°40'50" N, 37°14'53" E, *Aldasoro & Alarcón 9979* (BC)	KF028814	KM189331	KF028853	KM189230	KM189260
	KF028815	KM189332	KF028854	KM189231	KM189261
	KF028816	KM189333	KF028855	KM189232	KM189262
	KM189330		KM189229		KM189263
Ethiopia, Agere Maryan, Yirga Chefé Road, 14/08/2009, 2434m, 6º 04' 54" N, 38º 14' 33" E, *Aldasoro & Alarcón 10060* (BC)	KF028820	KF028825	KF028859	KF028863	KM189275
	KF028821	KF028826	KF028860	KF028864	KM189276
	KF028822	KM189339	KF028861	KF028865	KM189277
	KF028823	KM189340	KF028862	KM189238	KM189278
	KF028824			KM189239	KM189279

Ethiopia, Harenna Forest, Negele to Goba Road, 19/08/2009, 2182m, 6º 42' 03" N, 39º 43' 35" E, *Aldasoro & Alarcón 10322* (BC)	KF028827	KF028831	KF028866	KF028870	KM189284
	KF028828	KF028832	KF028867	KF028871	KM189285
	KF028829	KM189341	KF028868	KM189240	KM189286
	KF028830		KF028869		KM189287
Ethiopia, Harenna Forest, Negele to Goba Road, 19/08/2009, 1939m, 6º 39' 3"N, 39º 43' 57"E, *Aldasoro & Alarcón 10317* (BC)	KF028833		KF028872		KM189290
Uganda, Mt. Elgon 31/08/2010, 2230 m, 1º 19' 44" N, 34º, 24' 58" E, *Sánchez-Meseguer & Aldasoro 69* (BC)	KF028834	KM189345	KF028873	KM189244	KM189292
	KF028835	KM189346	KF028874	KM189245	KM189293
	KF028836	KM189347	KF028875	KM189246	KM189294
	KF028837	KM189348	KF028876	KM189247	KM189295
	KF028838	KM189349	KF028877	KM189248	KM189296
	KF028839	KM189350	KF028878	KM189249	KM189297
	KM189342	KM189351	KM189241	KM189250	KM189298
	KM189343	KM189352	KM189242	KM189251	KM189299
	KM189344		KM189243		KM189300
Uganda, Ruwenzori Mts, 27/08/2010, 2599 m, 0º 21' 35" N, 29º 58' 20" E, *Sánchez-Meseguer & Aldasoro18* (BC)	KF028840	KF028846	KF028879	KF028885	KM189309
	KF028841	KM189353	KF028880	KM189252	KM189310
	KF028842	KM189354	KF028881	KM189253	KM189311
	KF028843	KM189355	KF028882	KM189254	KM189312
	KF028844	KM189356	KF028883	KM189255	KM189313
	KF028845	KM189357	KF028884	KM189256	KM189314
Kenya, Aberdare Mts, 03/09/2010, 2467 m, 0º 45' 52" N, 36º 44' 35" E, *Sánchez-Meseguer & Aldasoro103* (BC)	KF028847	KF028852	KF028886	KF028891	KM189321
	KF028848	KM189358	KF028887	KM189257	KM189322
	KF028849	KM189359	KF028888	KM189258	KM189323
	KF028850	KM189360	KF028889	KM189259	KM189324
	KF028851		KF028890		KM189325
Kenya, K2 N. Cherangani Hills Kaibwibich. IS377	XXX		XXX		XXX
Tanzania, Mbeya: Rungwe District. Livingstone Mountains; foot trail above Bumbigi. IS379	XXX		XXX		XXX
Tanzania, Mbeya: Rungwe District. Road between Igoma and Kitulo on south slope of Mporoto Mountains. IS378	XXX		XXX		XXX
Tanzania, Tukuyu district. IS382	XXX		XXX		XXX

Tanzania, Rungwe District. Mwakeleli, vincinity of Mwatesi River. IS381	XXX	XXX	XXX
Uganda, Mount Elgon, Sasa trail. IS376	XXX	XXX	XXX
Rwanda, Gikongoro Prefecture: Route Butare-Cyangugu, vers km 60. IS384	XXX	XXX	XXX
Burundi, Teza. IS386	XXX	XXX	XXX
Malawi, Misuku District, Sllindi Forest, Misuku Hills. IS 383	XXX	XXX	XXX

Table S2.3. Specific primer pairs used in the amplification and sequencing of plastid markers in *C. eminii*.

Primer name's	Primer Sequence (5'–3')	Author primer's
***trn*S^{GCU} -F**	GCC GCT TTA GTC CAC TCA GC	Hamilton, 1999
***trn*G^{UCC} -R**	GAA CGA ATC ACA CTT TTA CCA C	Hamilton, 1999
***pet*B^{1365}-F**	TTG ACY CGT TTT TAT AGT TTA	Löhne and Borsch, 2005
***pet*D^{738}-R**	AAT TTA GCY CTT AAT ACA GG	Löhne and Borsch, 2005
***rpl*32-F**	CAG TTC CAA AAA AAC GTA CTT C	Shaw *et al.*, 2007
***trnL*^{UAG}-R**	CTG CTT CCT AAG AGC AGC GT	Shaw *et al.*, 2007

Table S2.4. Hierarchical analysis of molecular variance (AMOVA) for *C. eminii* based on AFLP data at different levels.

**AMOVA for all populations**						
**Groupings**	**Source of variation**	**d.f.**	**Sum of squares**	**Variance components**	**Percentage of variation**	**Φ Statistics**
**1 group**	Among populations	5	2966.483	51.70776	40.42	-
	Within populations	55	4192.042	76.21894	59.58	Φ_{ST}= 0.4042
	**Total**	**60**	**7158.525**	**127.92670**		
**2 groups: Eastern Rift-Western Rift:**	Among groups	1	712.698	5.17812	3.98	Φ_{CT}= 0.0398
1) **Western Rift**: Gifta, Dembecha, Mt. Elgon and Ruwenzori Mts.	Among populations	4	2253.785	48.69257	37.43	Φ_{SC}= 0.3898
**2) Eastern Rift**: Agere Maryan, Harenna Forest and Aberdare Mts.	Within populations	55	4192.042	76.21894	58.59	Φ_{ST}= 0.4141
	**Total**	**60**	**7158.525**	**130.08963**		
**3 groups:**	Among groups	2	1443.203	3.30884	2.57	Φ_{CT}=

Groupings	Source of variation	d.f.	Sum of squares	Variance components	Percentage of variation	Φ Statistics
						0.0257
1. Abyssinian Plateau (Gifta and Dembecha)	Among populations	3	1523.280	49.05.202	38.15	Φ_{SC}= 0.3916
2. Agere Maryan, Harenna Forest and Aberdare Mts	Within populations	55	4192.042	76.21894	59.28	Φ_{ST}= 0.4072
3. Elgon and Rwenzori Mts.	**Total**	**60**	**7158.525**	**128.57979**		
**4 groups:**						
1. Abyssinian Plateau (Gifta and Dembecha)	Among groups	3	2187.769	14.13032	10.89	Φ_{CT}= 0.1089
2. Agere Maryan, Harenna Forest and Aberdare Mts.	Among populations	2	778.714	39.34721	30.34	Φ_{SC}= 0.3405
3. Elgon Mts.	Within populations	55	4192.042	76.21894	58.77	Φ_{ST}= 0.4123
4. Rwenzori Mts	**Total**	**60**	**7158.525**	**129.69647**		
**AMOVA for Eastern Rift populations**						
**Groupings**	**Source of variation**	**d.f.**	**Sum of squares**	**Variance components**	**Percentage of variation**	**Φ Statistics**
**1 group in the Eastern Rift:**	Among populations	2	778.714	38.93272	32.87	
Harar massif (Agere Maryan, Harenna Forest) and Aberdare Mts.	Within populations	21	1669.869	79.51757	67.13	Φ_{ST}= 0.3287
	**Total**	**23**	**2448.583**	**118.45029**		
**2 groups in the Eastern Rift**	Among groups	1	395.356	4.91574	4.09	Φ_{CT}= 0.0409
1. Harar massif: Agere Maryan and Harenna Forest	Among populations	1	383.358	35.87003	29.82	Φ_{SC}= 0.3109
2. Aberdare Mts.	Within populations	21	1669.869	79.51757	66.10	Φ_{ST}= 0.3390
	**Total**	**23**	**2448.583**	**120.30335**		
**AMOVA for Western Rift populations**						
**Groupings**	**Source of variation**	**d.f.**	**Sum of squares**	**Variance components**	**Percentage of variation**	**Φ Statistics**
**1 group Western:**	Among populations	2	1475.071	55.03159	42.59	
Debre Markos, Elgon and Rwenzori Mts.	Within populations	34	2522.173	74.18155	57.41	Φ_{ST}= 0.42590
	**Total**	**36**	**3997.243**	**129.21314**		
**2 groups in the Western Rift**	Among groups	1	730.505	-11.89280	-9.42	Φ_{CT}= -0.0942
1. Abyssinian plateau: Debre Markos	Among populations	1	744.565	63.99118	50.67	Φ_{SC}= 0.4631
2. Elgon and Rwenzori Mts.	Within populations	34	2522.173	74.18155	58.74	Φ_{ST}= 0.4126
	**Total**	**36**	**3997.243**	**126.27993**		
**AMOVA for Ethiopian plateaus populations**						
**Groupings**	**Source of variation**	**d.f.**	**Sum of squares**	**Variance components**	**Percentage of variation**	**Φ Statistics**
**2 groups Eastern Plateau - Western Plateau:**	Among groups	1	726.589	12.54985	9.28	Φ_{CT}= 0.0928
1. Abyssinian Plateau: Gifta and Dembecha	Among populations	1	383.358	34.89585	25.81	Φ_{SC}= 0.2845
2. Harar Plateau: Agere Maryan and Harenna Forest	Within populations	30	2633.083	87.76944	64.91	Φ_{ST}= 0.3509
	**Total**	**32**	**3743.030**	**135.21515**		

Table S2.5. Migration routes supported by Bayes Factor comparisons with a cut off value >3 using Bayesian stochastic search variable selection (BSSVS, Lemey *et al*., 2009).

Migration routes	Bayes Factor
Harenna Forest - Aberdare Mts.	7.6760
Rwenzori - Mt. Elgon	5.3979
Mt. Elgon - Rwenzori	4.6847
Aberdare Mts - Harenna Forest	4.0291
Rwenzori - Southern Mountain sky-islands	3.8762
Rwenzori - Abyssinian massif	3.6395
Mt.Elgon - Southern Mountain sky-islands	3.3288
Southern Mountain sky-islands - Rwenzori	3.1858
Southern Mountain sky-islands - Mt. Elgon	3.0084

Table S2.6. Pairwise F_{ST} between populations of *C. eminii* with nuclear data (below) and geographic distances in km (above).

	Gifta	Dembecha	Agere Maryan	Harenna Forest	Mt. Elgon	Rwenzori Mts.	Aberdare Mts.
**Gifta**	-	28	546	545.9	1091.84	1398.95	1115.13
**Dembecha**	0.0801	-	520.37	517.78	1080.55	1396.78	1098
**Agere Maryan**	0.2157	0.1593	-	177.87	675.86	1112.88	611.1
**Harenna Forest**	0.3028	0.2352	0.1706	-	836.87	1287.61	735.28
**Mt. Elgon**	0.3555	0.2877	0.2985	0.3698	-	503.09	266.4
**Rwenzori Mts.**	0.3306	0.2627	0.2887	0.2174	0.3779	-	752
**Aberdare Mts.**	0.3041	0.2390	0.2058	0.2247	0.3302	0.3142	-

Figure S2.1.

Nested analysis of the Platycodoneae dataset. Numbers above branches indicate mean ages and numbers below branches indicate Bayesian PP. For more details see Mairal *et al.* 2015a.

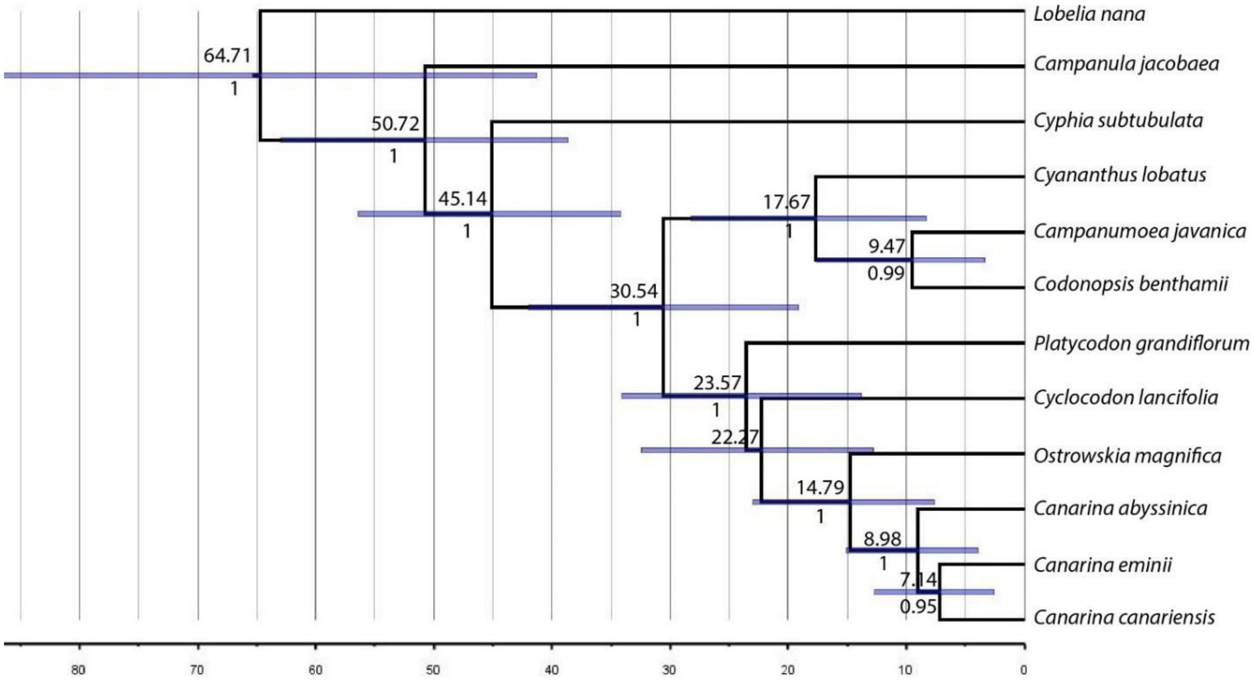

Figure S2.2. Histograms showing the Bayesian clustering of individuals within populations (STRUCTURE) for a) K=2 and b) K=3. Colours represent the proportion of individual membership to each inferred Bayesian group. c) The estimated probability of the likelihood function according to the Evanno method for STRUCTURE analyses of *C. eminii*. Maximum Δ*K* values correspond to the presumed true number of K cluster**s.**

a)

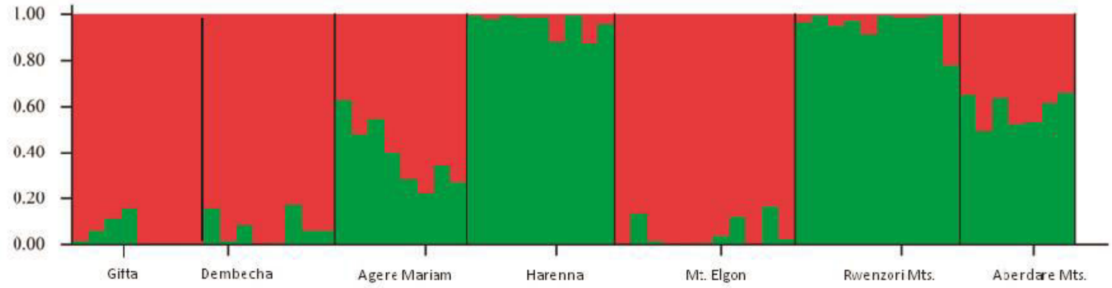

b)

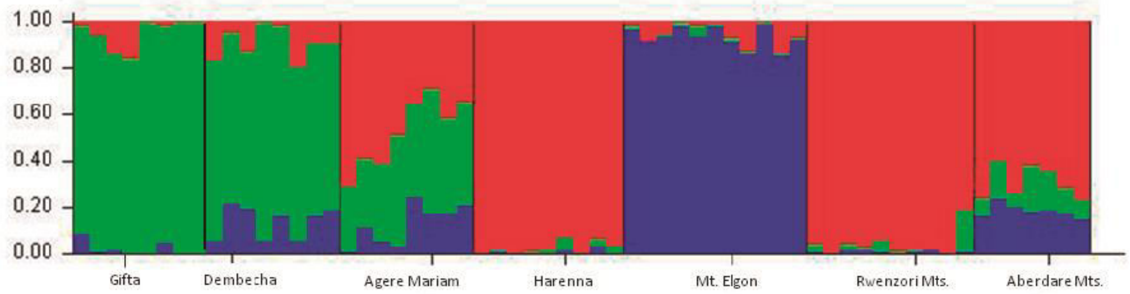

c)

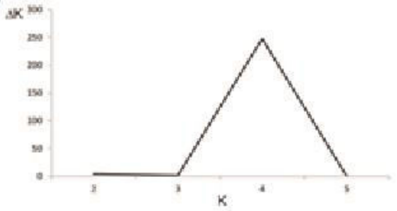

K groups	L(K)	stdev	L'(K)	L"(K)	[L"K]	delta K
**1**	-22408,89	1,319				
**2**	-20282,01	153,241	2126,88	-664,81	664,81	4,338
**3**	-18819,94	112,03	1462,07	-146,942	146,94	1,311
**4**	-17504,812	5,574	1315,127	-1380,602	1380,6	247,661
**5**	-17570,287	636,51	-65,475	488,148	488,15	0,766

Figure S2.3. Neighbor tree (A) and neighbor-net (B) for the AFLP data for individuals of *C. eminii*. Numbers are bootstrap values from neighbor-joining analysis using Nei-Li distances over 1000 bootstrap replicates. To root the neighbor tree, we used a population of *C. abyssinica* collected in Ethiopia (ab).

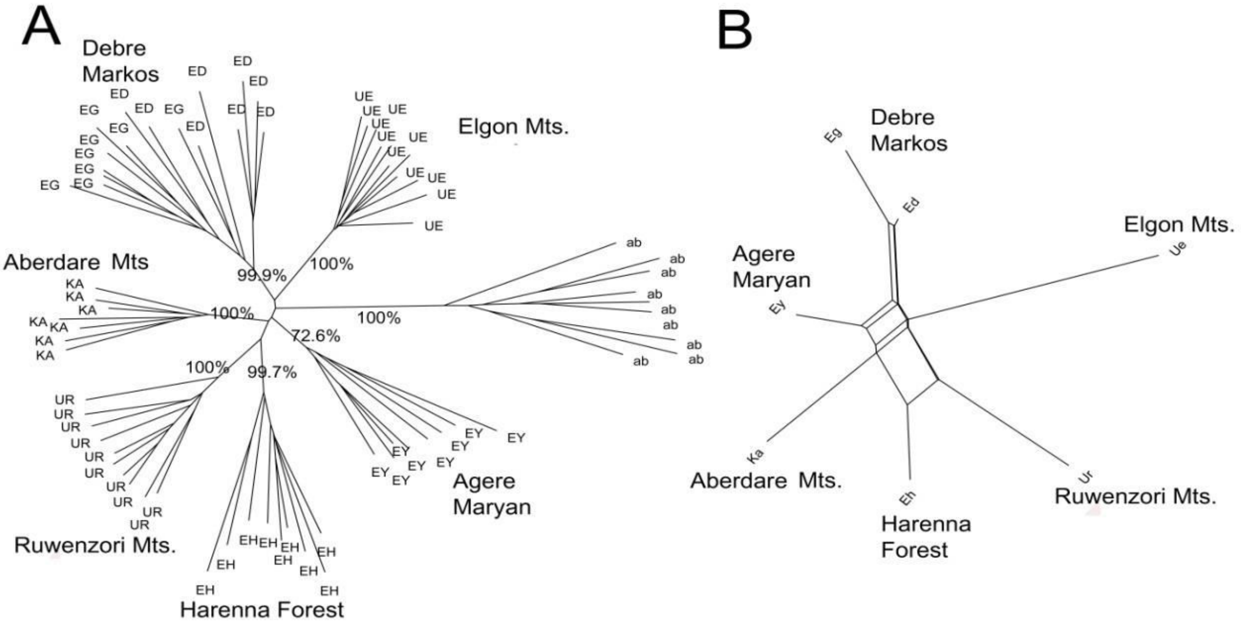

Figure S2.4. Principal coordinates analysis of AFLP data for individuals of *C. eminii.*

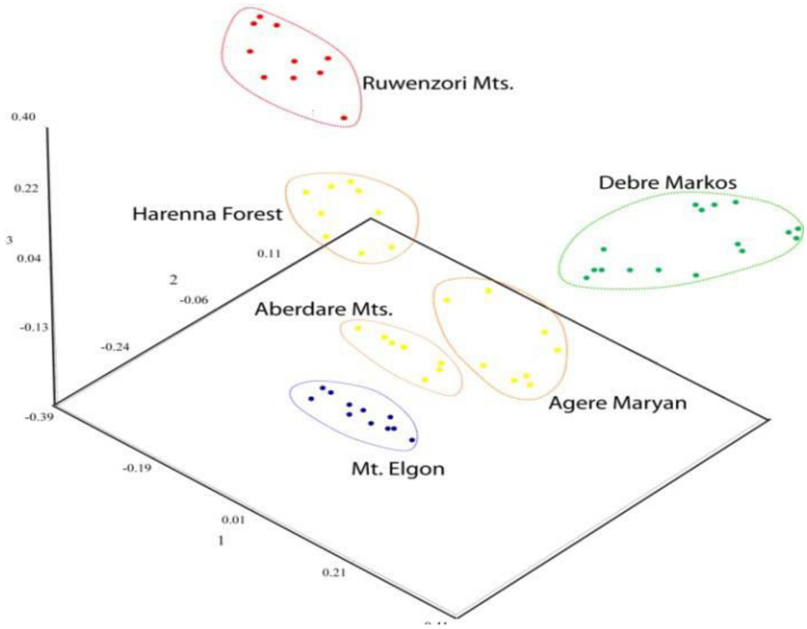

Figure S2.5. Regression plot among pairwise F_{ST} and logarithms of geographical distances. Acronyms are E: Elgon Mt., R: Rwenzori, D: Debre Markos (Gifta and Dembecha), Y: Agere Maryan, A: Abredare Mts., H: Harenna Forest.

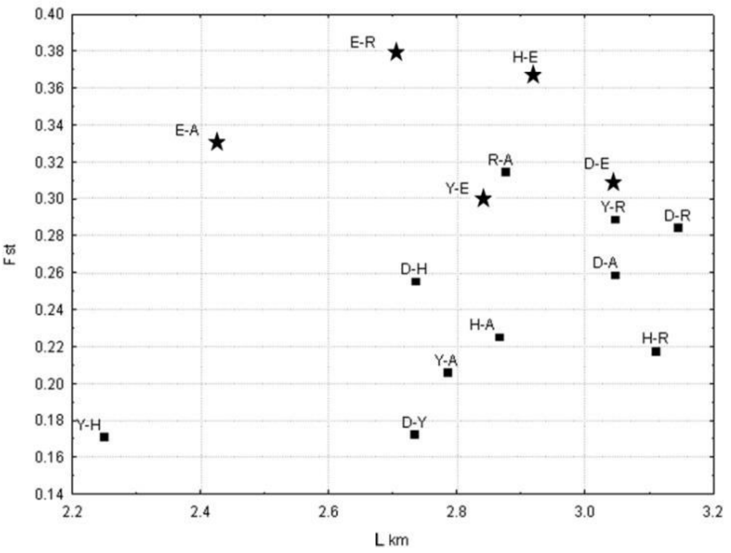

Figure S2.6. Distances intervals for *C. eminii* populations in SPAGeDi analysis (dotted lines show the 95% confidence level).

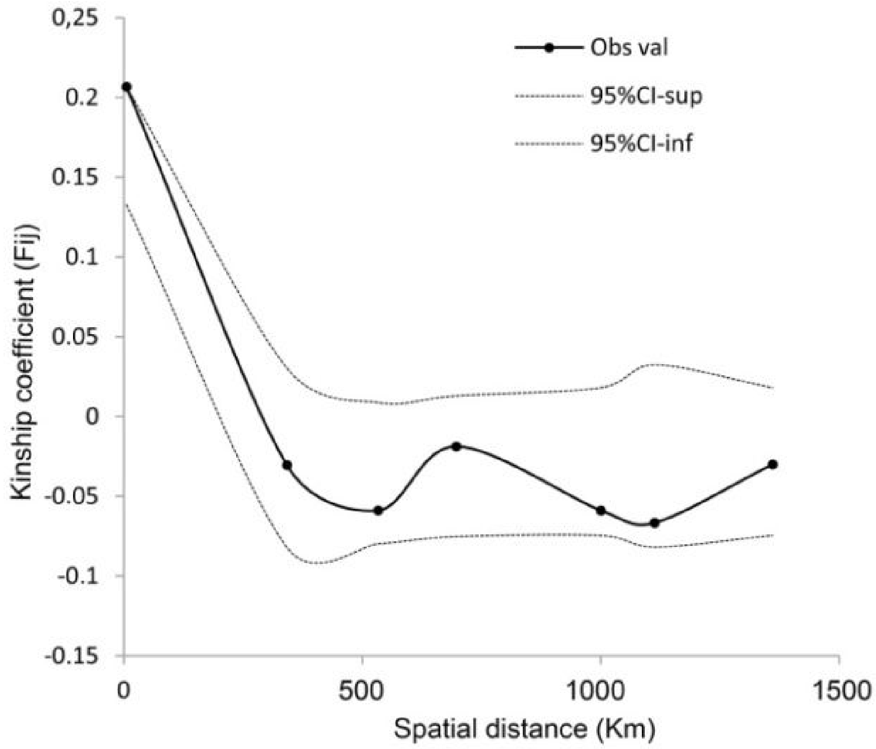

Appendix S3 Supplemental references

Arctander, P., Johansen, C. & Coutellec-Vreto, M.A. Phylogeography of three closely related African bovids (tribe Alcelaphini). *Molecular Biology and Evolution.* **16**, 1724-1739 (1999).

Belay, G. & Mori, A. Intraspecific phylogeographic mitochondrial DNA (D-loop) variation of Gelada baboon, *Theropithecus gelada*, in Ethiopia. *Biochemical Systematics and Ecology*. **34**, 554-561 (2006).

Braginets, O.P., Minakawa, N., Mbogo, C.M. & Ya, G. Population genetic structure of the African malaria mosquito *Anopheles funestus* in Kenya. *The American journal of tropical medicine and hygiene*. **69**, 303-308 (2003).

Colangelo, P. *et al*. A mitochondrial phylogeographic scenario for the most widespread African rodent, *Mastomys natalensis*. *Biological Journal of the Linnean Society*. **108**, 901-916 (2013).

Derero, A., Gailing, O. & Finkeldey. R. Maintenance of genetic diversity in *Cordia africana* Lam., a declining forest tree species in Ethiopia. *Tree Genetics & Genomes*. **7**, 1-9 (2010).

Dubach, J. *et al*. Molecular genetic variation across the southern and eastern geographic ranges of the African lion, *Panthera leo*. *Conservation Genetics*. **6**, 15-24 (2005).

Evanno, G., Regnaut, S. & Goudet, J. Detecting the number of clusters of individuals using the software STRUCTURE: a simulation study. *Molecular Ecology*. **14**, 2611-2620 (2005).

Evans, B.J., Bliss, S.M., Mendel, S.A. & Tinsley, R.C. The Rift Valley is a major barrier to dispersal of African clawed frogs (*Xenopus*) in Ethiopia. *Molecular Ecology*. **20**, 4216-4230 (2011).

Field, L.M. *et al*. Analysis of genetic variability in *Anopheles arabiensis* and *Anopheles gambiae* using microsatellite loci. *Insect Molecular Biology*. **8**, 287-297 (1999).

Girman, D.J. *et al*. Patterns of population subdivision, gene flow and genetic variability in the African wild dog (*Lycaon pictus*). *Molecular Ecology*. **10**, 1703-1723 (2001).

Hall, T.A. BioEdit: a user-friendly biological sequence alignment editor and analysis program for Windows 95/98/NT. *Nucleic acids symposium series*. **41**, 95-98 (1999).

Hamilton, M. Four primers pairs for the amplification of chloroplast intergenic regions with intraspecific variation. *Molecular Ecology*. **8**, 521-523 (1999).

Kadu, C.A.C., *et al*. Divergent pattern of nuclear genetic diversity across the range of the Afromontane *Prunus africana* mirrors variable climate of African highlands. *Annals of Botany*. **111**, 47-60 (2013).

Katoh, K., Kuma, K., Toh, H. & Miyata, T. MAFFT version 5: improvement in accuracy of multiple sequence alignment. *Nucleic Acids Research*. **33**, 511-518 (2005).

Kelchner, S.A. The Evolution of Non-Coding Chloroplast DNA and Its Application in Plant Systematics. *Annals of the Missouri Botanical Garden*. **87**, 482 (2000).

Krafsur, E.S. Population structure of the tsetse fly *Glossina pallidipes* estimated by allozyme, microsatellite and mitochondrial gene diversities. *Insect Molecular Biology*. **11**, 37-45 (2002).

Lehmann, T. *et al*. The Rift Valley complex as a barrier to gene flow for *Anopheles gambia*e in Kenya. *Journal of Heredity*. **90**, 613-621 (1999).

Lehmann, T. *et al*. Brief communication. The Rift Valley complex as a barrier to gene flow for *Anopheles gambiae* in Kenya: the mtDNA perspective. *Journal of Heredity*. **91**, 165-168 (2000).

Lehmann, T. *et al*. Population Structure of *Anopheles gambiae* in Africa. *Journal of Heredity*. **94**, 133-147 (2003).

Löhne, C. & Borsch, T. Molecular evolution and phylogenetic utility of the *petD* group II intron: a case study in basal angiosperms. *Molecular Biology and Evolution*, **22**. 317-332 (2005).

Matthee, C.A. & Robinson, T.J. Molecular phylogeny of the springhare, *Pedetes capensis*, based on mitochondrial DNA sequences. *Molecular Biology and Evolution*- **14**, 20-29 (1997).

Muchugi, A. *et al*. Genetic structuring of important medicinal species of genus *Warburgia* as revealed by AFLP analysis. *Tree Genetics & Genomes*. **4**, 787-795 (2008).

Nicolas, V. *et al*. Phylogeographic structure and regional history of *Lemniscomys striatus* (Rodentia: Muridae) in tropical Africa. *Journal of Biogeography*. **35**, 2074-2089 (2008).

Omondi, S.F. *et al*. Genetic Diversity and Population Structure of *Acacia senegal* (L) Willd. in Kenya. *Tropical Plant Biology*. **3**, 59-70 (2010).

Pitra, C., Hansen, A.J., Lieckfeldt, D. & Arctander, P. An exceptional case of historical outbreeding in African sable antelope populations. *Molecular Ecology*. **11**, 1197-1208 (2002).

Pokorny, L., Oliván, G., Shaw, A. Phylogeographic patterns in two southern hemisphere species of *Calyptrochaeta* (Daltoniaceae, Bryophyta). *Systematic Botany*. **36**, 542-553 (2011).

Posada, D. jModelTest: phylogenetic model averaging. *Molecular Biology and Evolution*. **25**, 1253-1256 (2008).

Rambaut A., Drummond A.J. & Suchard M. Tracer v1. 6. http://tree.bio.ed.ac.uk/software/tracer/ (2007)

Rambaut, A. & Drummond, A. FigTree v1.3.1. http://tree. bio. ed. ac. uk/software/figtree (2009).

Ronquist, F. & Sanmartín, I. Phylogenetic methods in biogeography. *Annual Review of Ecology, Evolution, and Systematics*. **42**, 441 (2011).

Sertse, D., Gailing, O., Eliades, N.G. & Finkeldey, R. Anthropogenic and natural causes influencing population genetic structure of *Juniperus procera* Hochst. ex Endl. in the Ethiopian highlands. *Genetic Resources and Crop Evolution*. **58**, 849-859 (2011).

Shaw, J., Lickey, E.B., Schilling, E.E. & Small, R.L. Comparison of whole chloroplast genome sequences to choose noncoding regions for phylogenetic studies in angiosperms: the tortoise and the hare III. *American Journal of Botany*. **94**, 275-288 (2007).

Silvestrini, M., *et al*. Genetic diversity and structure of Ethiopian, Yemen and Brazilian *Coffea arabica* L. accessions using microsatellites markers. *Genetic Resources and Crop Evolution*. **54**, 1367-1379 (2007).
